# In Vitro Quantification of Fluoride Levels in Commonly Consumed Foods and Beverages in Tirupati, India

**DOI:** 10.7759/cureus.107935

**Published:** 2026-04-29

**Authors:** Keerthi Gandhasiri, Prathyusha P, Archana Vadla, Sri Lakshmi Kedari, Haritha M, Revuru Aditya Srinivas

**Affiliations:** 1 Pediatric Dentistry, Mamata Dental College and Hospital, Khammam, IND; 2 Pediatric and Preventive Dentistry, CKS Teja Dental College and Hospital, Tirupati, IND; 3 Pedodontics and Preventive Dentistry, G.Pulla Reddy Dental College & Hospital, Kurnool, IND; 4 Pedodontics and Preventive Dentistry, CKS Teja Dental College and Hospital, Tirupati, IND; 5 Periodontics and Implantology, CKS Teja Dental College and Hospital, Tirupati, IND; 6 Pediatric and Preventive Dentistry, Dr. N.T.R University of Health Sciences, Vijayawada, IND

**Keywords:** bottled beverages, dental caries, dental fluorosis, fluoride, fruit juices, soft drinks

## Abstract

Background: Fluoride has both protective and harmful effects. Optimal daily intake prevents dental caries, while excess may lead to dental fluorosis. In India, bottled beverages widely consumed by children are a significant yet inconsistent source of dietary fluoride.

Objectives: The study aimed to quantify fluoride levels in popular soft drinks, fruit juices, vegetables, and dairy products available in Tirupati and to assess their potential risk-benefit profile for oral health.

Materials and methods: This in vitro analytical study included 60 samples categorized into four groups: carbonated beverages, fruit juices, vegetables, and dairy products (n = 15 per group). Fluoride concentration was measured using a calibrated ion-selective electrode method. Data were analyzed using one-way analysis of variance (ANOVA) and Tukey’s post hoc test, with statistical significance set at p < 0.01.

Results: The mean fluoride concentration was highest in vegetables (1.42 ± 0.08 ppm), followed by dairy products (1.06 ± 0.05 ppm), fruit juices (0.90 ± 0.03 ppm), and carbonated beverages (0.82 ± 0.03 ppm). Among individual samples, spinach showed the highest fluoride level (1.52 ppm), while carbonated beverages exhibited the lowest values (0.80-0.83 ppm). A statistically significant difference in fluoride levels was observed among the groups (p < 0.001), with vegetables and dairy products demonstrating significantly higher concentrations than beverages.

Conclusion: Fluoride levels in commonly consumed foods and beverages show considerable variation, with potential implications for cumulative dietary fluoride exposure. Monitoring these sources is important to maintain a balance between caries prevention and the risk of fluorosis.

Recommendations: Regulation of fluoride levels in packaged foods and beverages is essential. Mandatory labeling of fluoride content should be implemented to aid informed decision-making. In low-fluoride environments, carefully calibrated supplementation may be warranted, while communities exposed to high dietary fluoride require monitoring to safeguard against fluorosis.

## Introduction

Dental caries remains one of the most prevalent oral diseases in India, affecting nearly 50%-85% of children across different regions. Fluoridation has proven effective in reducing caries, though fluoride levels in India vary due to environmental diversity [1]. Fluoride, a naturally occurring mineral, prevents demineralization, promotes remineralization, and exhibits antibacterial effects [2]. It is administered systemically or topically, with water fluoridation being the most cost-effective approach. Major systemic sources in children include drinking water, carbonated beverages, and juices [3]. The American Academy of Pediatrics (1986) recommended 0.05-0.07 mg F/kg body weight/day for optimal protection, while intakes above 0.10 mg F/kg risk fluorosis [4]. WHO also endorsed fluoride use for caries prevention.

Excessive fluoride exposure, however, poses health hazards. WHO recommends a limit of 1.5 mg/L in drinking water [5]. Chronic exposure may result in dental fluorosis, skeletal changes, and non-skeletal effects such as gastrointestinal, neurological, endocrine, renal, and reproductive disorders [6]. High intake has also been linked to thyroid dysfunction, miscarriages, congenital defects, and cancers. Fluoride accumulation in cerebrospinal fluid can adversely affect the brain [7]. Monitoring dietary fluoride intake is essential, as restricting a single source does not ensure safety [8].

With changes in lifestyle and increasing urbanization, children are exposed to higher amounts of fluoride from packaged foods and beverages, particularly during critical stages of tooth development [9]. In recent years, the consumption of sweetened and acidic beverages has also increased significantly [10]. Drinks with a pH below 4.0 can contribute to dental erosion by directly dissolving enamel and dentin, leading to sensitivity, functional impairment, and esthetic concerns in severe cases [11-13]. Studies report that dental erosion affects 10% to 80% of children, with primary teeth being more susceptible [14].

In addition to their erosive potential, these commonly consumed foods and beverages may serve as significant and variable sources of dietary fluoride. While optimal fluoride intake plays a crucial role in caries prevention, both deficient and excessive exposure may result in adverse outcomes, including increased risk of dental fluorosis. However, region-specific data on fluoride content in commonly consumed dietary items in India remain limited. Therefore, it is essential to quantify fluoride levels in these sources to better understand their contribution to total fluoride intake and associated oral health implications. In this context, the present study aims to evaluate the fluoride concentration in commonly consumed foods and beverages available in Tirupati, Andhra Pradesh.

## Materials and methods

Following institutional ethical approval (CKS/PEDO/20-21/001), this in vitro analytical study was conducted to estimate the fluoride content of commonly consumed foods and beverages in Tirupati, Andhra Pradesh, India. The study included four major categories of dietary samples: carbonated beverages, fruit juices, vegetables, and dairy products, selected based on their widespread consumption and contribution to the local diet across different age groups.

A pilot study was conducted to calibrate the methodology, standardize the fluoride ion-selective electrode, and ensure reproducibility of results. Sample size estimation was performed using one-way analysis of variance (ANOVA) (F-test) for four groups, with a significance level (α) of 0.05, desired power of 80%, and a large effect size (Cohen’s f = 0.50), yielding an estimated requirement of 60 samples (~15 per group). Accordingly, a total of 60 samples were included and divided into four groups of 15 samples each.

Each group comprised three different product types, with five independent samples collected for each product (n = 5 per product). The groups were categorized as follows: (1) carbonated beverages, (2) fruit juices, (3) vegetables, and (4) dairy products. For each category, commonly available items were selected from local retail outlets. Only fresh, sealed, and unexpired samples were included, while compromised or previously opened samples were excluded.

Sample preparation was performed according to standard protocols. Beverages and fruit juices were degassed using a magnetic stirrer for 30 minutes and diluted in a 1:1 ratio with buffer solution [8]. Vegetables were thoroughly washed, oven-dried at 80°C, homogenized, and sieved; cooked samples were prepared by boiling in deionized water. Dairy products were centrifuged at 3000 rpm, and the supernatant was neutralized before analysis [15]. All samples were coded, stored in polyethylene containers at room temperature, and protected from light until analysis.

The pH of each sample was measured in triplicate using a calibrated digital pH meter standardized with buffer solutions of pH 4 and 7. Fluoride estimation was performed using a fluoride ion-selective electrode (Adwa AD8000, Romania), calibrated with sodium fluoride standards ranging from 0.1 to 10 ppm. Each sample was analyzed in duplicate, and the mean value was recorded. Fluoride concentration was calculated using the formula $F = \frac{R \times 50}{W}$, where F represents fluoride concentration (ppm), R is the electrode reading, and W is the sample weight or volume.

The multiplication factor accounts for dilution and standardization using the total ionic strength adjustment buffer (TISAB), which maintains constant ionic strength and pH, ensuring accurate electrode response. This approach allows normalization of fluoride measurements across different sample types, thereby improving analytical reliability and comparability. Each product type was considered an independent sample unit. The mean of duplicate measurements was used for statistical analysis. Data normality was assessed using the Shapiro-Wilk test, and homogeneity of variance was evaluated using Levene’s test before applying ANOVA.

Data were analyzed using SPSS version 20.0 (IBM Corp., Armonk, NY, USA). One-way ANOVA was employed for intergroup and intragroup comparisons, and statistical significance was set at p < 0.01.

## Results

The fluoride concentration varied across different sample categories. Among carbonated beverages, carbonated beverages 1 and 2 showed similar fluoride levels (0.83 ppm), while carbonated beverage 3 exhibited slightly lower concentration (0.80 ppm), resulting in a group mean of 0.82 ± 0.03 ppm. In the fruit juice category, apple juice demonstrated the highest fluoride concentration (0.92 ppm), followed by orange juice (0.91 ppm) and watermelon juice (0.88 ppm), with an overall mean of 0.90 ± 0.03 ppm. Vegetables exhibited comparatively higher fluoride concentrations, with spinach showing the highest value (1.52 ppm), followed by carrot (1.39 ppm) and potato (1.34 ppm), yielding a group mean of 1.42 ± 0.08 ppm. Among dairy products, milk showed the highest fluoride concentration (1.08 ppm), followed by cheese (1.06 ppm) and yogurt (1.05 ppm), with a mean value of 1.06 ± 0.05 ppm. Overall, vegetables demonstrated the highest fluoride concentrations, followed by dairy products, while fruit juices and carbonated beverages exhibited comparatively lower levels. Statistical analysis revealed significant differences between groups (p < 0.001) (Table 1). 

**Table 1:** Mean fluoride concentration (ppm) among different sample groups One-way ANOVA followed by Tukey’s post hoc test was used for intergroup comparison. *p < 0.05 = significant; **p < 0.001 = highly significant ANOVA, analysis of variance.

Test parameters	Sample	Mean (ppm)	Standard deviation	Intra-group p-value	Intra-group F-value	Inter-group p-value
Carbonated beverages	Carbonated beverage 1	0.83	0.03	0.226	2862.000	0.000**
	Carbonated beverage 2	0.83	0.03			
	Carbonated beverage 3	0.80	0.2			
	Total	0.82	0.03			
Juices	Apple juice	0.92	0.03	0.025*		
	Orange juice	0.91	0.02			
	Watermelon juice	0.88	0.03			
	Total	0.90	0.03			
Vegetables	Potato	1.34	0.02	0.000**		
	Spinach	1.52	0.01			
	Carrot	1.39	0.06			
	Total	1.42	0.08			
Milk	Milk	1.08	0.08	0.000**		
	Cheese	1.06	0.03			
	Yogurt	1.05	0.05			
	Total	1.06	0.05			

**Figure 1:**
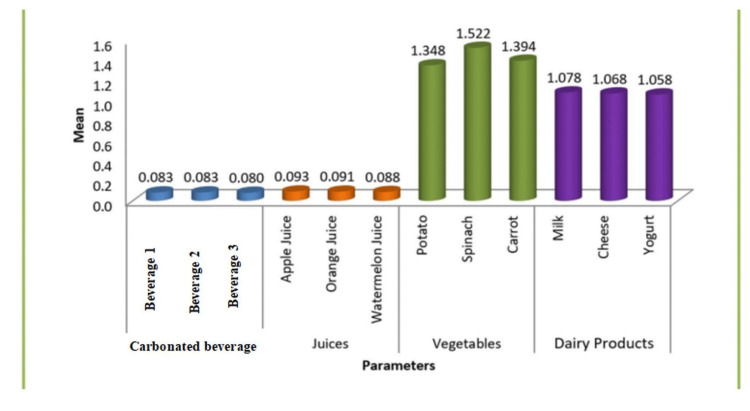
Mean intergroup comparisons of samples One-way ANOVA revealed a statistically significant difference in mean fluoride levels among the four groups of samples (p < 0.001). ANOVA, analysis of variance.

One-way ANOVA revealed a statistically significant difference in mean fluoride levels among the four groups of samples (p < 0.001). Post hoc Tukey’s test demonstrated that the mean fluoride concentration in vegetables (1.348-1.522 ppm) was significantly higher (p < 0.001) than dairy products (1.058-1.078 ppm), fruit juices (0.088-0.093 ppm), and carbonated beverages (0.083 ppm). Similarly, dairy products also showed significantly greater fluoride content than both fruit juices and carbonated beverages (p < 0.001). However, no significant difference was observed between fruit juices and soft drinks (p > 0.05), indicating comparably low fluoride levels in these beverages (Figure 1).

Tukey’s post hoc analysis revealed statistically significant differences in fluoride concentrations between most of the groups. Vegetables demonstrated significantly higher fluoride levels than dairy products, fruit juices, and carbonated beverages (p < 0.001). Similarly, dairy products showed significantly higher fluoride concentrations than both fruit juices and carbonated beverages (p < 0.001). However, no statistically significant difference was observed between fruit juices and carbonated beverages (p > 0.05), indicating comparable fluoride levels in these two groups (Table 2).

**Table 2:** One-way ANOVA followed by Tukey’s post hoc test was used for pairwise comparisons. *p < 0.05 = statistically significant ANOVA, analysis of variance.

Comparison	Mean difference (ppm)	p-value
Vegetables vs dairy	0.36	<0.001
Vegetables vs fruit juices	0.52	<0.001
Vegetables vs carbonated beverages	0.60	<0.001
Dairy vs fruit juices	0.16	<0.001
Dairy vs carbonated beverages	0.24	<0.001
Fruit juices vs carbonated beverages	0.08	>0.05

## Discussion

Fluoride remains one of the most effective agents for caries prevention, with an optimal intake of 0.05-0.07 mg/kg body weight, while higher levels increase the risk of dental fluorosis [15]. In the present study, fluoride concentrations varied significantly across different food categories, with vegetables (1.42 ± 0.08 ppm) showing the highest levels, followed by dairy products (1.06 ± 0.05 ppm), fruit juices (0.90 ± 0.03 ppm), and carbonated beverages (0.82 ± 0.03 ppm). These findings indicate that dietary sources beyond drinking water may serve as potential contributors to fluoride exposure, particularly among children with high consumption of packaged foods and beverages.

The higher fluoride levels observed in vegetables in the present study are consistent with previous reports attributing this to environmental factors such as soil composition and irrigation water. Similar variability has been reported by Cantoral et al. [8] and Kiritsy et al. [16], who documented fluoride levels ranging from 0.09 to 0.56 ppm in food products, while higher concentrations have also been observed in certain regions due to geochemical variations [17,18].

In the present study, dairy products demonstrated moderate fluoride levels, with milk showing slightly higher concentrations than yogurt and cheese. This is in agreement with findings by Gupta et al. [19], who reported higher fluoride levels in milk relative to other dairy products. In addition to fluoride content, dairy products may contribute to caries prevention through calcium, phosphorus, and casein phosphopeptides, which enhance remineralization.

Fluoride concentrations in fruit juices and carbonated beverages in the present study were comparatively lower and showed no significant difference between the two groups. These findings are consistent with previous studies conducted in India and internationally. For example, Chowdhury et al. [20] reported fluoride levels of 0.09-0.21 mg/L in fruit juices, while Thippeswamy et al. [21] observed similar ranges in bottled beverages. International studies by Jimenez Farfan et al. [22] and Liu et al. [23] also demonstrated considerable variability in fluoride concentrations across beverages, supporting the findings of the present study.

The observed variability in fluoride content across different food categories and studies highlights the influence of multiple factors, including regional water fluoride levels, soil composition, food processing methods, and manufacturing practices. This variability underscores the importance of monitoring dietary fluoride sources to maintain a balance between its cariostatic benefits and the risk of fluorosis.

Although the present study provides valuable insights into fluoride content in commonly consumed foods and beverages, it is important to interpret the findings cautiously. As this was an in vitro analysis, actual fluoride intake and exposure could not be assessed, and therefore, the results represent potential rather than definitive contributions to total fluoride intake.

From a public health perspective, the findings emphasize the need for improved regulatory oversight and quality control of fluoride levels in commercially available products. Encouraging transparent labeling of fluoride content may enhance consumer awareness and support clinicians in making informed recommendations. In regions with low fluoride exposure, carefully monitored supplementation may be beneficial, whereas areas with higher exposure require regular surveillance to prevent fluorosis.

This study has certain limitations. The sample size was relatively modest and limited to a single geographic region, which may affect generalizability. Additionally, variations in manufacturing processes and individual consumption patterns were not evaluated. Future research should focus on larger, multicentric studies incorporating dietary assessments and biomarkers of fluoride exposure to better understand the cumulative impact on oral health.

## Conclusions

Within the scope of this in vitro investigation, noticeable variation in fluoride levels was observed among different categories of commonly consumed foods and beverages. The highest concentrations were identified in vegetables, followed by dairy products, whereas fruit juices and carbonated beverages exhibited relatively lower levels. These findings suggest that multiple dietary sources, beyond beverages alone, may potentially contribute to overall fluoride exposure, although actual intake was not assessed in the present study.

The observed variability underscores the need for careful evaluation and monitoring of dietary fluoride sources to maintain a balance between its protective effects against dental caries and the risk of excessive exposure. Regular monitoring of fluoride levels in commonly consumed foods and beverages, along with improved labeling practices, may help in better assessment and management of fluoride exposure. Further research involving larger sample sizes and assessment of actual consumption patterns is necessary to better understand the cumulative impact on oral health.
